# Potent neutralization of SARS‐CoV‐2 including variants of concern by vaccines presenting the receptor‐binding domain multivalently from nanoscaffolds

**DOI:** 10.1002/btm2.10253

**Published:** 2021-09-09

**Authors:** Peter J. Halfmann, Ana Castro, Kathryn Loeffler, Steven J. Frey, Shiho Chiba, Yoshihiro Kawaoka, Ravi S. Kane

**Affiliations:** ^1^ Influenza Research Institute, Department of Pathobiological Sciences, School of Veterinary Medicine University of Wisconsin Madison Wisconsin USA; ^2^ School of Chemical and Biomolecular Engineering Georgia Institute of Technology Atlanta Georgia USA; ^3^ Division of Virology, Department of Microbiology and Immunology, Institute of Medical Science University of Tokyo Tokyo Japan; ^4^ Center for Global Viral Infections National Center for Global Health and Medicine Tokyo Japan

**Keywords:** COVID‐19, nanoparticle, neutralization, receptor‐binding domain, SARS‐CoV‐2, vaccine, variants of concern

## Abstract

The persistence of the global severe acute respiratory syndrome coronavirus 2 (SARS‐CoV‐2) pandemic has brought to the forefront the need for safe and effective vaccination strategies. In particular, the emergence of several variants with greater infectivity and resistance to current vaccines has motivated the development of a vaccine that elicits a broadly neutralizing immune response against all variants. In this study, we used a nanoparticle‐based vaccine platform for the multivalent display of the receptor‐binding domain (RBD) of the SARS‐CoV‐2 spike (S) protein, the primary target of neutralizing antibodies. Multiple copies of RBD were conjugated to the SpyCatcher‐mi3 protein nanoparticle to produce a highly immunogenic nanoparticle‐based vaccine. RBD‐SpyCatcher‐mi3 vaccines elicited broadly cross‐reactive antibodies that recognized the spike proteins of not just an early isolate of SARS‐CoV‐2, but also three SARS‐CoV‐2 variants of concern as well as SARS‐CoV‐1. Moreover, immunization elicited high neutralizing antibody titers against an early isolate of SARS‐CoV‐2 as well as four variants of concern, including the delta variant. These results reveal the potential of RBD‐SpyCatcher‐mi3 as a broadly protective vaccination strategy.

## 1 INTRODUCTION

The novel severe acute respiratory syndrome coronavirus 2 (SARS‐CoV‐2) that emerged in late 2019 quickly spread globally and has already led to more than 3 million deaths worldwide.^1^ Numerous vaccine candidates are therefore being developed to provide protection against SARS‐CoV‐2, including nucleic acid‐based vaccines, viral vector‐based vaccines, subunit vaccines, and inactivated vaccines.^2^ Most of the vaccines in development aim to elicit a protective immune response that targets the spike (S) protein of SARS‐CoV‐2. The receptor‐binding domain (RBD) of the trimeric S protein, which initiates infection by binding to the host cell receptor angiotensin‐converting enzyme 2 (ACE2),^3^ is the primary target of neutralizing antibodies elicited by vaccination or infection. These antibodies are able to neutralize the virus by either binding to the receptor‐binding motif to directly inhibit binding to ACE2 or by binding to the RBD in a manner that locks it in an unstable state, leading to dissociation of the trimer.

Although several vaccines have been approved for clinical use and have demonstrated high effectiveness against the original strain of the virus, the recently emerged “variants of concern” are better able to escape neutralization by vaccine‐induced humoral immunity, leading to a decrease in vaccine potency. The emergence of variants has motivated the design and testing of booster shots that can provide protection against these new circulating strains. While this is a reasonable near‐term approach, it would be desirable to develop a vaccine that would provide broad protection against emerging SARS‐CoV‐2 variants.

While S‐targeting vaccines in current use are based on the full‐length S protein, vaccines based on the RBD,^4^, ^5^, ^6^, ^7^, ^8^ the primary target of neutralizing antibodies, are worth exploring. Moreover, parts of the RBD are conserved, not just between the SARS‐CoV‐2 variants, but also between SARS‐CoV‐2 and SARS‐CoV‐1. Antibodies binding to these conserved regions have already been shown to neutralize SARS‐CoV‐2 as well as SARS‐CoV‐1 pseudoviruses.^9^, ^10^ Lederer et al.^4^ recently compared two RBD vaccine platforms—an mRNA vaccine and recombinant RBD formulated with AddaVax, an MF59‐like adjuvant—and reported that the mRNA vaccines were superior at eliciting SARS‐CoV‐2 specific germinal center B‐cell responses. This work, however, used monomeric recombinant RBD; in contrast, several groups have reported robust protective immune responses upon vaccination with RBDs presented multivalently from nanoparticle scaffolds^5^, ^7^, ^8^ and at least one such candidate is in clinical trials.^11^ Tan et al.^5^ used SpyCatcher/SpyTag chemistry for the assembly of the SARS‐CoV‐2 RBD on SpyCatcher003‐mi3 nanoparticles and showed that a prime‐boost regimen elicited strong neutralizing antibody responses in mice and pigs that were superior to those in convalescent human sera. Cohen et al.^7^ designed mosaic nanoparticles co‐displaying SARS‐CoV‐2 RBD along with RBDs from other animal betacoronaviruses that elicited antibodies with cross‐reactive recognition of heterologous RBDs. Kang et al. designed three different RBD‐conjugated nanoparticles and reported higher neutralizing antibody titers against authentic SARS‐CoV‐2 virus for the resulting antisera relative to those for mice immunized with monomeric RBD.^6^ Recently, Sanders et al.^8^ showed that macaque immunization with a multimeric SARS‐CoV‐2 RBD nanoparticle elicited cross‐neutralizing antibody responses against SARS‐CoV‐2, the variants of concern (B.1.1.7, P.1, and B.1.351), SARS‐CoV‐1, and bat coronaviruses.

We wanted to test if this exciting result showing broad protection could be generalized to other scaffolds displaying RBD multivalently. We therefore designed and tested vaccine constructs based on SpyCatcher‐mi3 nanoscaffolds, which have been used to display a variety of different antigens through SpyTag‐SpyCatcher conjugation,^12^ including the SARS‐CoV‐2 RBD.^5^, ^6^ By addition of a SpyTag to the C‐terminus of RBD, we were able to irreversibly attach multiple copies of the RBD to each SpyCatcher‐mi3 particle. The efficacy of the vaccine construct was then tested against a panel of variants. Immunization studies demonstrated the production of a strong and broadly cross‐reactive humoral response against SARS‐CoV‐2 and SARS‐CoV‐1. Furthermore, the immunization elicited high neutralizing antibody titers, not just against an early isolate of SARS‐CoV‐2, but also against four important “variants of concern” including the delta variant (B.1.617.2).

## 2 RESULTS AND DISCUSSION

**FIGURE 1:**
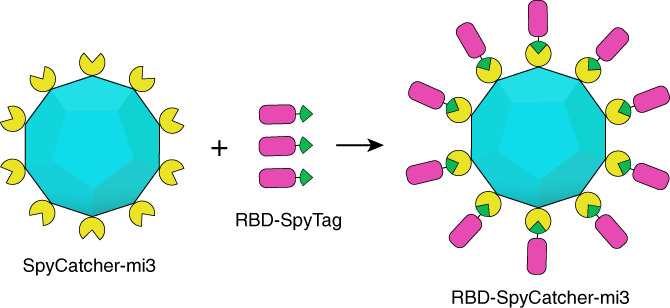
Schematic illustrating the generation of receptor‐binding domain (RBD)‐SpyCatcher‐mi3 conjugates by the reaction of RBD‐SpyTag with Spycatcher‐mi3 nanoparticles. (RBD: magenta; SpyTag: green; SpyCatcher: yellow; mi3: cyan)

**FIGURE 2:**
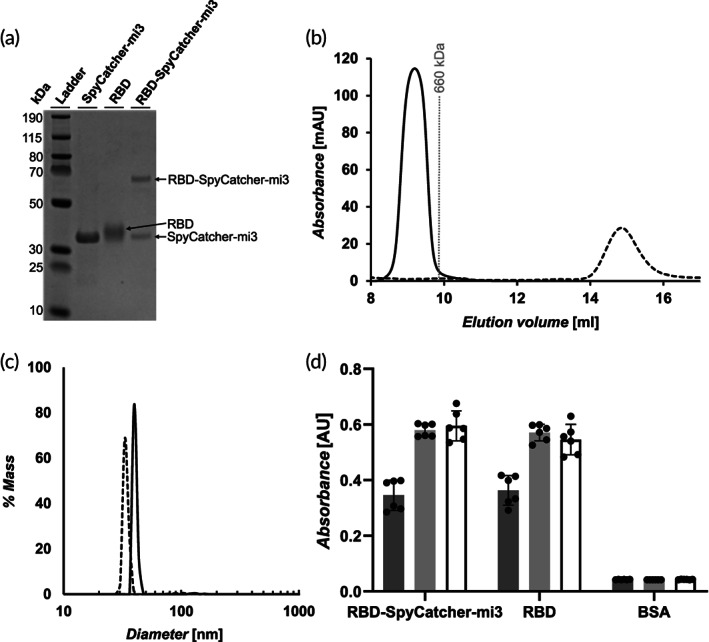
Characterization of receptor‐binding domain (RBD) and RBD‐SpyCatcher‐mi3. (A). Characterization of SpyCatcher‐mi3, RBD, and RBD‐SpyCatcher‐mi3 by SDS‐PAGE. The unprocessed gel is shown in Figure S3. (B). Size exclusion chromatography curves for RBD (dashed line) and RBD‐SpyCatcher‐mi3 (solid line). The gray line represents the peak elution volume of the molecular weight standard thyroglobulin. The column void volume is 7.2 ml. (C). Characterization of the RBD‐SpyCatcher‐mi3 (solid line) and SpyCatcher‐mi3 (dashed line) by dynamic light scattering. (D). Characterization of the binding of ACE‐2‐Fc (dark gray), CR3022 (light gray), and S309 (white) to RBD, RBD‐SpyCatcher‐mi3, and BSA (control) by enzyme‐linked immunosorbent assay (ELISA) (mean ± SD, *n* = 6: two assays with three technical replicates)

To begin, we produced protein nanoparticles that use SpyTag‐SpyCatcher technology for the multivalent display of RBD (Figure 1). The SpyCatcher‐mi3 scaffold is based on a mutated aldolase protein from a thermophilic bacterium fused to the SpyCatcher protein that self‐assembles into a dodecahedral 60‐mer virus‐like particle.^12^ The SpyCatcher allows for the conjugation of SpyTagged proteins through the formation of an isopeptide bond, making it a versatile and efficient platform. The SpyCatcher‐mi3 was expressed in BL21 (DE3) competent *Escherichia coli* cells. After cell lysis, Spycatcher‐mi3 was purified with a CaptureSelect C‐tag affinity column and size exclusion chromatography (SEC). Purity was assessed by sodium dodecyl sulphate–polyacrylamide gel electrophoresis (SDS‐PAGE) (Figure 2A).

Next, we generated an RBD construct to be conjugated to SpyCatcher‐mi3. The SpyTag sequence (AHIVMVDAYKPTK) was inserted at the C‐terminus of the RBD (amino acids 319‐541 of SARS‐CoV‐2 S protein) followed by a 6xHis‐tag. The SpyTag enables conjugation to SpyCatcher‐mi3, while the 6xHis‐Tag enables purification by immobilized metal affinity chromatography (IMAC). The construct was expressed in Expi293F cells. Secreted protein was purified from the media using IMAC, then purified further with SEC to remove aggregates and other impurities.

The RBD‐SpyTag was mixed with SpyCatcher‐mi3 overnight to generate RBD‐SpyCatcher‐mi3. Each particle contained approximately 30 RBD monomers, as determined by SDS‐PAGE (Figure S1). Characterization by SDS‐PAGE showed two bands: the upper band at ~65 kDA corresponds to a SpyCatcher‐mi3 monomer conjugated to an RBD monomer, and the lower band at ~34 kDA corresponds to a mi3 monomer alone (Figure 2A). The RBD‐SpyCatcher‐mi3 was further characterized by SEC and dynamic light scattering (DLS). SEC chromatograms of RBD‐SpyCatcher‐mi3 and RBD (Figure 2B) show the expected shift based on the large difference in molecular weight between the RBD monomer (25 kDa) and RBD‐SpyCatcher‐mi3 (~2.8 MDa). The curve for RBD‐SpyCatcher‐mi3 also only contains a single peak, which shows that there is very little unreacted RBD. DLS indicated a diameter of 40 nm for RBD‐SpyCatcher‐mi3 and a diameter of 34 nm for the SpyCatcher‐mi3 nanoparticle alone (Figure 2C). The increase in diameter for RBD‐SpyCatcher‐mi3 is consistent with the addition of an RBD layer to the outside of the nanoparticle and with the expected RBD diameter of 3 nm. To confirm that the RBD remained properly folded after conjugated to SpyCatcher‐mi3, we characterized the binding of ACE2‐Fc (an Fc fusion protein of the ACE2 receptor), and the RBD‐specific antibodies S309 (a cross‐neutralizing antibody)^10^ and CR3022 (a conformation‐dependent antibody)^13^ to RBD and RBD‐SpyCatcher‐mi3 by enzyme‐linked immunosorbent assay (ELISA) (Figure 2D). ELISA results confirmed the ability of both RBD and RBD‐SpyCatcher‐mi3 to be recognized by all three proteins, confirming the proper folding of important epitopes.

**FIGURE 3:**
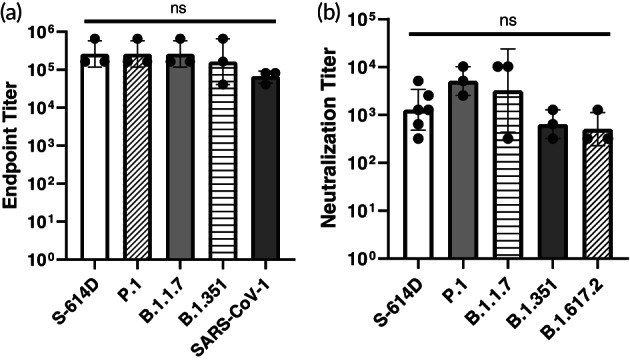
Antibody response to receptor‐binding domain (RBD)‐SpyCatcher‐mi3. (A). Antibody endpoint titers of sera from mice immunized with a prime and boost of RBD‐SpyCatcher‐mi3 against the spike proteins of an early isolate of SARS‐CoV‐2 (S‐614D), SARS‐CoV‐2 variants P.1, B.1.1.7, and B.1.351 and SARS‐CoV‐1 (geometric mean with geometric SD, *n* = 3 against all S protein: sera from 3 mice). ns = not statistically significant, determined by a one‐way analysis of variance (ANOVA) and Tukey post‐hoc multiple comparisons between groups (*α* = 0.05). (B). Viral neutralization titers for sera from mice immunized with RBD‐SpyCatcher‐mi3 (geometric mean with geometric SD, *n* = 6 against S‐614D and *n* = 3 against all other viral strains: sera from three mice). Endpoint titers using 2‐fold diluted sera were expressed as the reciprocal of the highest dilution that completely prevented cytopathic effects. ns = not statistically significant, determined by a ANOVA and Tukey post‐hoc multiple comparisons between groups (α = 0.05)

Next, we immunized mice with the RBD‐SpyCatcher‐mi3 vaccines to evaluate the immune response. RBD‐SpyCatcher‐mi3 mixed with AddaVax, a vaccine adjuvant consisting of an oil‐in‐water nano‐emulsion, was administered to mice (*n* = 3), followed by a boost 25 days later. Mice were bled before the boost (25 days after the initial immunization) then terminally bled (47 days after the initial immunization) to collect sera and characterize the breadth of the antibody response. High titers against an early isolate of SARS‐CoV‐2 (S‐614D) and three variants of concern—P.1, B.1.1.7, and B.1.351—were seen after a single immunization with RBD‐SpyCatcher‐mi3 (Figure S2). A second immunization boosted antibody titers against these SARS‐CoV‐2 variants and also elicited high antibody titers against SARS‐CoV‐1 (Figure 3A and Table 1). Importantly, we also observed high neutralizing antibody titers against the early isolate of SARS‐CoV‐2 as well as 4 SARS‐CoV‐2 variants of concern—P.1, B.1.1.7, B.1.351, and B.1.617.2 (Figure 3B and Table 1). RBD‐SpyCatcher‐mi3 demonstrated significantly higher neutralization titers against an early isolate of SARS‐CoV‐2 compared to those previously reported for an AddaVax‐adjuvanted monomeric RBD.^4^ There was no significant difference in the endpoint antibody titers and neutralizing antibody titers against the different strains (Figure 3A,B).

**TABLE 1:** Antibody responses to receptor‐binding domain (RBD)‐SpyCatcher‐mi3 after prime and boost in mice

	Spike IgG endpoint titer^a^									
	S‐614D		P.1		B.1.1.7		B.1.351		SARS‐CoV‐1	
Vaccine group	Geometric mean	Geometric SD factor	Geometric Mean	Geometric SD factor	Geometric mean	Geometric SD factor	Geometric Mean	Geometric SD factor	Geometric Mean	Geometric SD factor
SpyCatcher‐mi3	<20	—	<20	—	<20	—	<20	—	<20	—
RBD‐SpyCatcher‐mi3	260,080	2.23	260,080	2.23	260,080	2.23	163,840	4.00	65,020	1.49

	Neutralizing antibody titer^b^									
	S‐614D	P.1	B.1.1.7	B.1.351	B.1.617.2					
Vaccine group	Geometric Mean	Geometric Mean	Geometric Mean	Geometric Mean	Geometric Mean					
SpyCatcher‐mi3	<20	<20	<20	<20	<20					
RBD‐SpyCatcher‐mi3	1016	5120	3225	640	508					

^a^ Viral antibody endpoint titers against the SARS‐CoV‐2 and SARS‐CoV‐1 spike proteins (three animals in each group). Endpoint titers using 2‐fold diluted sera were expressed as the reciprocal of the highest dilution with an optical density at 490 nm cutoff value >0.15; sera were collected on day 47 after the initial immunization.

^b^ Viral neutralization titers (three animals in each group). Endpoint titers using 2‐fold diluted sera were expressed as the reciprocal of the highest dilution that completely prevented cytopathic effects; sera were collected on day 47 after the initial immunization.

We have compared these titers with some previously published reports for RBD‐based vaccines. We stress, however, that the assays used in the literature are not standardized and some reports have used different animal models. These differences in results must therefore be interpreted with caution. Tan et al. evaluated the neutralization potency of mice immunized with low doses of RBD‐SpyVLPs using a live SARS‐CoV‐2 virus neutralization assay.^5^ High neutralizing titers were seen, with the reciprocal of the dilution required for a 50% reduction in the number of plaques ranging from 450 to 2095 in C57BL/6 mice and 230 to 1405 in BALB/c mice. Negligible neutralizing antibody responses were seen in mice immunized with an equivalent amount of purified monomeric RBD. Lederer et al. immunized mice with two doses of RBD mRNA and also reported neutralizing antibody titers two‐logs higher than those for mice immunized with monomeric RBD protein.^4^ Kang et al. reported a similar enhancement in immunogenicity for RBD‐nanoparticle constructs compared to monomeric RBD.^6^ The reciprocal of the dilution required for 50% neutralization for sera from mice immunized with the RBD‐conjugated nanoparticles adjuvanted with AddaVax after the second boost was ~10^3^ and these neutralizing titers were 10 to 120‐fold greater than those for sera from animals immunized with the monomeric RBD. Thus, our neutralizing antibody titers after a prime and boost (Table 1), which represent the reciprocal of the highest dilution that completely prevented cytopathic effects, compare well with these other RBD‐based vaccine results against early isolates of SARS‐CoV‐2.

Recently, Saunders et al. immunized macaques with RBD‐ferritin nanoparticle conjugates (RBD‐scNP) and characterized neutralization efficacy against SARS‐CoV‐2 variants of concern. Two RBD‐scNP immunizations‐induced potent serum nAbs with 50% inhibitory reciprocal serum dilution neutralization titers ranging from 21,292 to 162,603. Neutralization was also reported against the variants B.1.1.7, B.1.351, and P.1. While efficacy against B.1.617.2 (delta) was not reported, we anticipate that RBD‐scNP sera would neutralize this variant, as we have shown for RBD‐SpyCatcher‐mi3 (Table 1).

We have also compared our neutralizing antibody titers against the variants of concern with those obtained from sera of immunized individuals. Tada et al. compared the neutralization titers of serum antibodies from individuals immunized with 3 U.S. FDA Emergency use authorization vaccines (BNT162b2, mRNA‐1273, and Ad26.COV2.S) using viruses pseudotyped with S proteins from SARS‐CoV‐2 variants.^14^ BNT162b2, mRNA‐1273, and Ad26.COV2.S sera neutralized virus pseudotyped with the D614G spike protein with average neutralizing antibody half‐maximal inhibitory concentration (IC_50_) titers of 695, 833, and 221 respectively. Neutralizing titers were lower—191, 208, and 30 respectively—for viruses pseudotyped with spike proteins from the Delta variant. Thus, our neutralizing antibody titers (Table 1) compare favorably with those obtained using these vaccine candidates that have been through clinical trials.

## 3 CONCLUSIONS

We have evaluated a vaccine candidate consisting of the SpyCatcher‐mi3 protein nanoparticle displaying the SARS‐CoV‐2 RBD. The RBD‐SpyCatcher‐mi3 retained the proper structure of binding epitopes following conjugation. This vaccine elicited very high levels of neutralizing antibodies in immunized mice against the original SARS‐CoV‐2 as well as three variants of concern. These studies strongly support the further testing of RBD‐based vaccines for clinical use as a vaccine that elicits broadly neutralizing antibodies.

Stamatatos et al.^13^ recently reported that the immunization of those previously infected with SARS‐CoV‐2 can significantly boost neutralizing antibody titers against all variants, with the neutralization attributed to antibodies targeting the RBD. In light of the robust and broadly protective responses seen in naïve mice by immunization with RBD‐SpyCatcher‐mi3, it will be interesting to characterize how the response is influenced by pre‐existing immunity due to infection or immunization. With an increasing percentage of the population already infected or vaccinated, characterization of the role of pre‐existing immunity will be an increasingly important consideration in vaccine design.

## 4 METHODS

### 4.1 Cloning of RBD constructs, S trimer, anti‐RBD antibodies, and SpyCatcher‐mi3

Construct 2019‐nCoV RBD‐SpyTag, encoding amino acids 319 to 541 from the SARS‐CoV‐2 S protein sequence (UniProt P0DTC2) followed by a GGSGG spacer, a SpyTag, and a 6xHis‐Tag, was optimized for expression in mammalian cells and synthesized by Gene Universal Inc. (Newark, DE).

Sequences encoding the light and heavy chains of the CR3022 antibody^15^ (retrieved from PDB 6 W41) and the S309 antibody^10^ (retrieved from PDB 6WPS) were cloned into the TGEX‐LC and TGEX‐HC vectors, respectively. To create the DNA construct for ACE2‐Fc, residues 1 to 615 of ACE2 were cloned into the TGEX‐HC vector. Codon optimization and DNA synthesis for all three constructs were carried out by Gene Universal Inc.

DNA encoding the SpyCatcher‐mi3 fusion protein^12^ was cloned into pET‐21a and synthesized by Gene Universal Inc. with no additional modifications. The DNA encoding the RBD constructs, ACE2‐Fc, CR3022, and S309 was transformed into 5‐α competent cells according to the manufacturer's recommendations. Transformed cells were grown at 37°C in 100 ml of 2xYT medium containing ampicillin. On the following day, the DNA was extracted and purified with an E.Z.N.A Plasmid Maxi Kit (Omega). The DNA coding for the mi3‐SpyCatcher was transformed into BL21(DE3) cells (New England Biolabs), which were grown overnight at 37°C and frozen as glycerol stocks for future use. All handling of recombinant DNA conformed to NIH guidelines.

### 4.2 Expression and purification of RBD, S, ACE2‐Fc, CR3022, and S309

RBD, ACE2‐Fc, CR3022, and S309 constructs were expressed in Expi293F suspension cells using the ExpiFectamine 293 transfection kit (A14524, Gibco) according to the manufacturer's protocol. Cells expressing RBD were harvested 4 days after transfection, centrifuged, and the supernatants were thoroughly dialyzed against IMAC Binding Buffer (100 mM Tris, 150 mM NaCl, 20 mM imidazole, pH 8.0).

Dialyzed proteins were poured over Ni‐NTA resin that had been pre‐equilibrated with 10 column volumes (CVs) of IMAC Binding Buffer. The resin was then washed with at least 20 CVs of IMAC Binding Buffer and eluted with 5 CVs of IMAC Elution Buffer (100 mM Tris, 150 mM NaCl, 400 mM imidazole, pH 8.0). The eluates were collected and concentrated using an Amicon spin filter (EMD MilliPore) with a 10 kDa MWCO. The concentrated RBD protein was further purified with a Superdex Increase 200 10/300 GL column for the removal of high molecular weight impurities.

Cells transfected with either ACE2‐Fc, CR3022, or S309 DNA were harvested 5 days after transfection and centrifuged to remove the cells and cell debris. The resulting supernatant was diluted in phosphate‐buffered saline (PBS) and loaded onto a MabSelect SuRe column (GE) to be purified according to the manufacturer's recommendations. The eluate containing the desired protein was then stored at 4°C for future use.

### 4.3 Expression and purification of SpyCatcher‐mi3

Cells transformed with the DNA coding for SpyCatcher‐mi3 were used for a 5 ml starter culture which was further scaled up (after growing for 12–16 h) to 1 L of 2xYT media containing kanamycin. Cells were grown at 37°C until the OD_600_ reached 0.8. The temperature was reduced to 22°C and isopropyl β‐D‐1‐thiogalactopyranoside (IPTG) was added to a final concentration of 0.5 mM. Cells were allowed to grow overnight before harvest and were then centrifuged at 7000*g* for 7 min.

Cell lysis and protein purification were performed according to the protocol described by Bruun et al.^12^ In brief, the cell pellet was resuspended in 20 ml of CaptureSelect Equilibration Buffer (25 mM Tris, 150 mM NaCl, pH 8.5) containing 2 μg of lysozyme, 125 units of benzonase, and half of a tablet of SigmaFast EDTA‐free (Sigma Aldrich) protease inhibitor cocktail. The mixture was incubated at room temperature for 1 h and then sonicated for 5 min with 5 s on, 5 s off pulses. Following sonication, the solution was centrifuged for 30 min at 17,000*g*, and the supernatant was poured over 5 ml of pre‐equilibrated CaptureSelect C‐tag Affinity Matrix (ThermoFisher Scientific). The resin was washed with 10 CVs of CaptureSelect Equilibration Buffer and eluted with CaptureSelect Elution Buffer (20 mM Tris, 2 M MgCl2, pH 8.5). All purification steps were performed at 4°C.

The eluate containing the protein of interest was dialyzed against 25 mM Tris, 150 mM NaCl, pH 8.5, overnight with a 50 kDa MWCO SpectraPor dialysis membrane (Repligen) and concentrated by spin filtration using a ViVaspin filter (Sartorius) with a 50 kDa MWCO. The concentrated protein was further purified by SEC using a Superdex Increase 200 10/300 GL column. Fractions corresponding to chromatogram peaks were analyzed by DLS, while fractions containing large amounts of aggregates were discarded. The remaining fractions were concentrated and stored at 4°C.

### 4.4 Conjugation of RBDs to SpyCatcher‐mi3 nanocages

Small scale reactions between RBD constructs and SpyCatcher‐mi3 were initially set up to determine optimal stoichiometric ratios. Mixtures were allowed to react overnight and the extent of RBD conjugation to the scaffold was determined by SDS‐PAGE. Reactions that showed a consumption of greater than 90% of RBD did not undergo any additional purification steps. Reactions that had lower yields were further purified by SEC using a GE Superdex 200 Increase 10/300 GL and eluate fractions containing the reaction product were concentrated. Sample purity and final RBD concentration were determined by SDS‐PAGE.

### 4.5 SDS‐PAGE

Protein samples were diluted in Nu‐PAGE lithium dodecyl sulfate (LDS) loading buffer (Invitrogen) to a final quantity of 1 μg. A 15 μL of protein samples and 2 μL of PageRuler Plus Prestained Protein Ladder were added to the wells of a 4%–12% Bis‐Tris gel (Invitrogen). Gels were run in MES‐SDS buffer at 120 V for 50 min, then stained with Imperial Protein Stain (ThermoFisher Scientific). After destaining, gels were imaged using the ChemiDoc MP imaging system and Image Lab 5.2.1 software (Bio‐Rad).

### 4.6 Dynamic light scattering

One**‐**hundred microliter samples of SpyCatcher‐mi3 or RBD‐SpyCatcher‐mi3 at a concentration of ~0.5 μg/μL were added to a UVette (Eppendorf). Dynamics software and a DynaPro NanoStar Dynamic Light Scattering detector were used to collect five acquisitions for each measurement. Acquisitions were averaged and results were displayed as % mass using the Isotropic Sphere model.

### 4.7 Analytical SEC

Samples of RBD and RBD‐SpyCatcher‐mi3, each containing 20 μg of RBD, were diluted in 1 ml of PBS. Samples were loaded into the sample loop which was then flushed with PBS to inject sample onto a Superdex 200 Increase 10/300 Column (Cytiva) using Unicorn 7 (Cytivia) control system. Protein was eluted with one column volume of PBS flowing at a flow rate of 0.5 ml/min. UV absorbance at 205, 210, and 280 nm was monitored.

### 4.8 Enzyme‐linked immunosorbent assay

Ninety‐six**‐**well plates were coated with 50 μL of RBD at 4 μg/ml and incubated at room temperature for 1 h. Wells were blocked with 100 μL of 5% (w/v) bovine serum albumin (BSA) diluted in PBS containing 0.1% tween‐20 (PBST) for 1 h. Blocking was followed by three washes with 100 μL of PBST and a 1‐h incubation with 50 μL of ACE2‐Fc, S309, or CR3022, diluted to a final concentration of 1 μg/ml in PBST with 1% BSA. The plates were washed three more times and incubated with horseradish peroxidase (HRP)‐conjugated anti‐human antibodies diluted 20,000 fold in PBST with 1% BSA. After a 1‐h incubation, plates were washed three more times and developed by adding 50 μL of 3,3′,5,5′‐tetramethylbenzidine (TMB) substrate. The reaction was quenched by adding 50 μL of stop solution (160 mM sulfuric acid) and the absorbance at 450 nm was measured.

### 4.9 Immunizations

All immunizations were performed by ProSci Inc. (Poway, CA). Three mice were immunized with a solution either 14 μg of RBD conjugated to mi3‐SpyCatcher or 16.8 μg of mi3‐SpyCatcher mixed with an equal volume of AddaVax adjuvant. On day 25, a boosting injection containing 20 μg of RBD antigen or 24 μg of mi3‐SpyCatcher mixed with AddaVax was administered. The mice were bled prior to the boost on day 25 and terminally bled on day 47. These immunizations and bleeds were carried out by ProSci Incorporated (Poway, CA) within their USDA licensed, registered and NIH/OLAW assured animal facility. All the protocols that included experimental animal procedures were carried out in accordance with the US Animal Welfare Act and approved by ProSci Incorporated's Institutional Animal Care and Use Committee.

### 4.10 Detection of anti‐RBD mouse antibodies

ELISAs were performed using recombinant spike antigens produced from codon‐optimized cDNA expressed in Expi293F cells (Thermo Fisher Scientific). Recombinant proteins with a C‐terminal HIS‐tag were purified by using TALON metal affinity resin.^16^ The recombinant spike antigen for SARS‐CoV‐1 strain Tor2 was purchased from Sino Biological. ELISA plates were coated overnight at 4°C with 50 μL of spike antigen at a concentration of 2 μg/ml in PBS. After blocking with PBS containing 0.1% Tween 20 (PBS‐T) and 3% milk powder, the plates with incubated in duplicate with heat‐inactivated serum diluted in PBS‐T with 1% milk powder. A mouse IgG secondary antibody conjugated with horseradish peroxidase (Invitrogen; 1:5000 dilution) was used for detection. Plates were developed with SigmaFast o‐phenylenediamine dihydrochloride solution (Sigma), and the reaction was stopped with the addition of 3 M hydrochloric acid. The absorbance was measured at a wavelength of 490 nm (OD_490_). Background absorbance measurements from pooled naïve mouse serum were subtracted from serum collected after immunization for each dilution. IgG antibody endpoint titers were defined as the highest serum dilution with an OD_490_ cut‐off value of 0.15.

### 4.11 Neutralization assay

The following viruses were used in the neutralization assays: SARS‐CoV‐2/UT‐NCGM02/Human/2020/Tokyo (S‐614D), hCoV‐19/Japan/TY7‐501/2021 (P.1), nCoV‐19/Japan/QHN001/2020 (B.1.1.7), hCoV‐19/USA/MD‐HP01542/2021 (B.1.351), and hCoV‐19/USA/WI‐UW‐5250/2021 (B.1.617.2). The assays were performed on VeroE6/TMPRSS2 cells obtained from the National Institute of Infectious Diseases, Japan.^17^ Viruses were incubated with the same volume of 2‐fold dilutions of heat‐inactivated serum for 30 min at 37°C. The antibody/virus mixture was added to confluent VeroE6/TMPRSS2 cells that were plated at 30,000 cells per well the day prior in 96‐well plates. The cells were incubated for 3 days at 37°C and then fixed and stained with 20% methanol and crystal violet solution. Virus neutralization titers were determined as the reciprocal of the highest serum dilution that completely prevented cytopathic effects.

### 4.12 Biosafety statement

SARS‐CoV‐2 was handled under biosafety level 3 agriculture (BSL‐3Ag) containment at the Influenza Research Institute with an approved protocol reviewed by approved the University of Wisconsin‐Madison's Institutional Biosafety Committee. The laboratory is designed to meet and exceed the standards outlined in *Biosafety in Microbiological and Biomedical Laboratories* (6th edition).

## CONFLICT OF INTEREST

The authors declare that there is no conflict of interest.

## AUTHOR CONTRIBUTIONS

**Peter Halfmann:** Data curation; formal analysis; investigation; writing‐review & editing. **Ana Castro:** Data curation; investigation; writing‐review & editing. **Kathryn Loeffler:** Data curation; formal analysis; investigation; writing ‐ original draft; writing‐review & editing. **Steven Frey:** Investigation; writing ‐ original draft; writing‐review & editing. **Shiho Chiba:** Investigation. **Yoshihiro Kawaoka:** Funding acquisition; supervision; writing‐review & editing. **Ravi Kane:** Conceptualization; funding acquisition; supervision; writing ‐ original draft; writing‐review & editing.

## Supporting information


**Figure S1** Characterization of RBD and mi3 conjugation stoichiometry by sodium dodecyl sulphate–polyacrylamide gel electrophoresis (SDS‐PAGE). Amount of unbound SpyCatcher‐mi3 monomer was compared to SpyCatcher‐mi3 standards (left side) to determine coverage of RBD on SpyCatcher‐mi3. ~50% of SpyCatcher‐mi3 monomers reacted, indicating each particle contained ~30 RBD proteins.
**Figure S2**. Antibody endpoint titers of sera from mice immunized with a single dose of RBDSpyCatcher‐mi3 against the spike proteins of an early isolate of SARS‐CoV‐2 (S‐614D) and SARS‐CoV‐2 variants B.1.1.7, B.1.351, and P.1 (geometric mean with geometric SD, *n* = 3 against all S protein: sera from three mice). ns = not statistically significant, determined by a one‐way analysis of variance (ANOVA) and Tukey post hoc multiple comparison between groups (α = 0.05).
**Figure S3**. Unprocessed SDS‐PAGE gel images. Cropped versions appear in (a) Figure 2a and (b) Figure S1.
**Supplementary Note 1.** SpyCatcher‐mi3 Sequence
**Supplementary Note 2**. RBD Sequence

## Data Availability

The data that support the findings of this study are available from the corresponding authors upon reasonable request.
