# Supplementary material for: Potent neutralization of SARS‐CoV‐2 including variants of concern by vaccines presenting the receptor‐binding domain multivalently from nanoscaffolds
Source: Bioeng Transl Med. 2021 Sep 9;6(3):e10253. doi: 10.1002/btm2.10253 (PMC8459632; doi:10.1002/btm2.10253)
Supplement: Supplementary file 1 — Figure S1 Characterization of RBD and mi3 conjugation stoichiometry by sodium dodecyl sulphate–polyacrylamide gel electrophoresis (SDS‐PAGE). Amount of unbound SpyCatcher‐mi3 monomer was compared to SpyCatcher‐mi3 standards (left side) to determine coverage of RBD on SpyCatcher‐mi3. ~50% of SpyCatcher‐mi3 monomers reacted, indicating each particle contained ~30 RBD proteins. Figure S2. Antibody endpoint titers of sera from mice immunized with a single dose of RBDSpyCatcher‐mi3 against the spike proteins of an early isolate of SARS‐CoV‐2 (S‐614D) and SARS‐CoV‐2 variants B.1.1.7, B.1.351, and P.1 (geometric mean with geometric SD, n = 3 against all S protein: sera from three mice). ns = not statistically significant, determined by a one‐way analysis of variance (ANOVA) and Tukey post hoc multiple comparison between groups (α = 0.05). Figure S3. Unprocessed SDS‐PAGE gel images. Cropped versions appear in (a) Figure 2a and (b) Figure S1. Supplementary Note 1. SpyCatcher‐mi3 Sequence Supplementary Note 2. RBD Sequence [file BTM2-6-e10253-s001.pdf]

Supporting Information

Potent neutralization of SARS-CoV-2 including variants of concern by vaccines presenting the receptor-binding domain multivalently from nanoscaffolds

Peter Halfmann[1,†], Ana Castro[2,†], Kathryn Loeffler[2,†], Steven J. Frey[2], Shiho Chiba[1], Yoshihiro Kawaoka[1,3,4*], Ravi S. Kane[2,*]

[1]Influenza Research Institute, Department of Pathobiological Sciences, School of Veterinary Medicine, University of Wisconsin, Madison, WI, 53711, USA

[2]School of Chemical & Biomolecular Engineering, Georgia Institute of Technology, Atlanta, Georgia, 30332, USA

[3]Division of Virology, Department of Microbiology and Immunology, Institute of Medical Science, University of Tokyo, Tokyo 108-8639, Japan

[4]Center for Global Viral Infections, National Center for Global Health and Medicine, Tokyo 162-8655, Japan

† These authors contributed equally

* To whom correspondence should be addressed:

yoshihiro.kawaoka@wisc.edu, ravi.kane@chbe.gatech.edu

Supplementary Figures

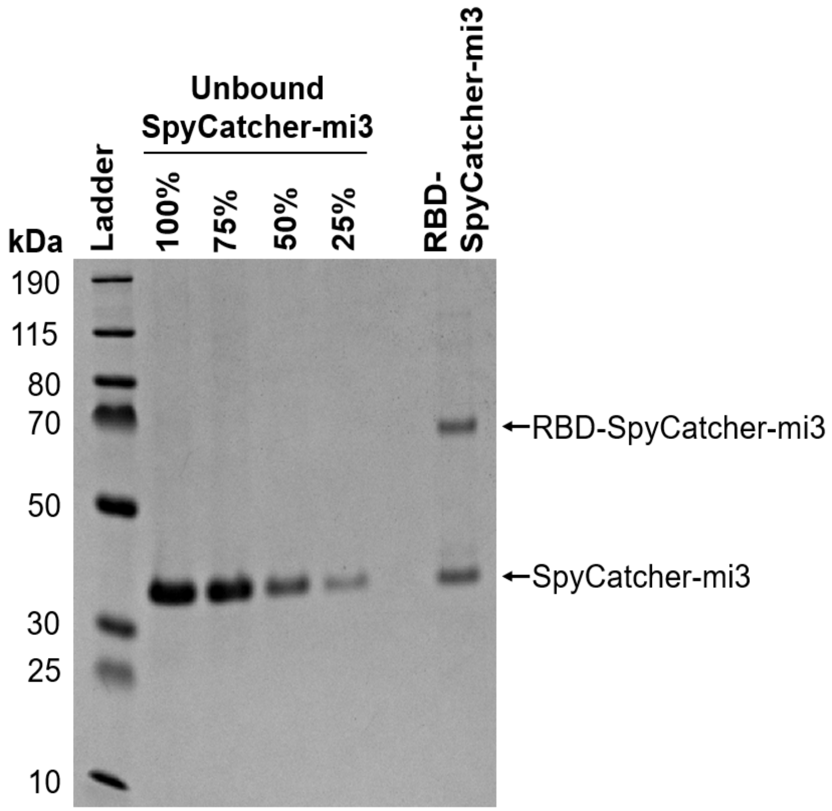

Supplementary Figure 1. Characterization of RBD and mi3 conjugation stoichiometry by SDS-PAGE. Amount of unbound SpyCatcher-mi3 monomer was compared to SpyCatcher-mi3 standards (left side) to determine coverage of RBD on SpyCatcher-mi3. ~50% of SpyCatcher-mi3 monomers reacted, indicating each particle contained ~30 RBD proteins.

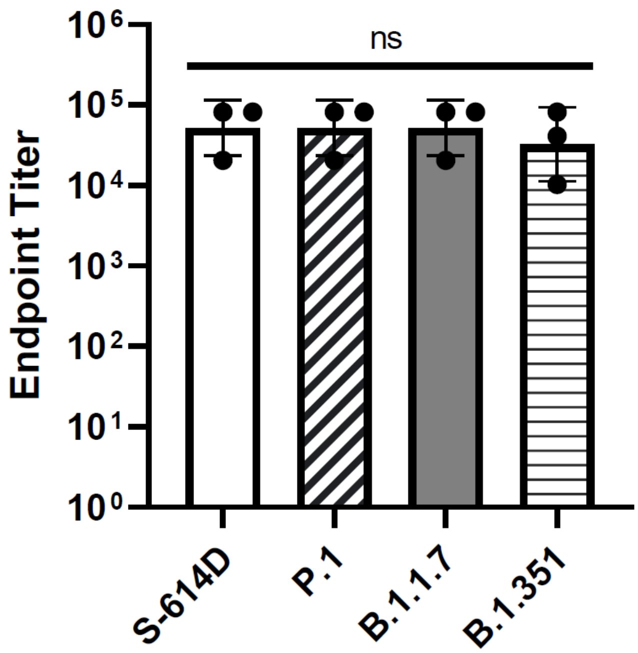

Supplementary Figure 2. Antibody endpoint titers of sera from mice immunized with a single dose of RBD-SpyCatcher-mi3 against the spike proteins of an early isolate of SARS-CoV-2 (S-614D) and SARS-CoV-2 variants B.1.1.7, B.1.351, and P.1 (geometric mean with geometric SD, n = 3 against all S protein: sera from 3 mice). ns = not statistically significant, determined by a one-way analysis of variance (ANOVA) and Tukey post-hoc multiple comparison between groups (α = 0.05).

a

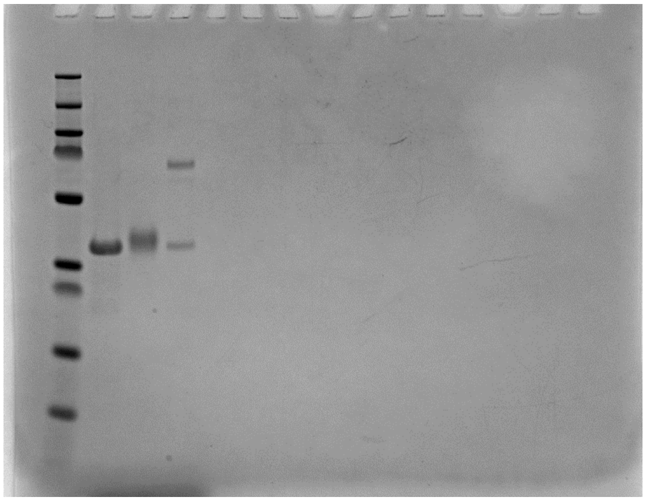

b

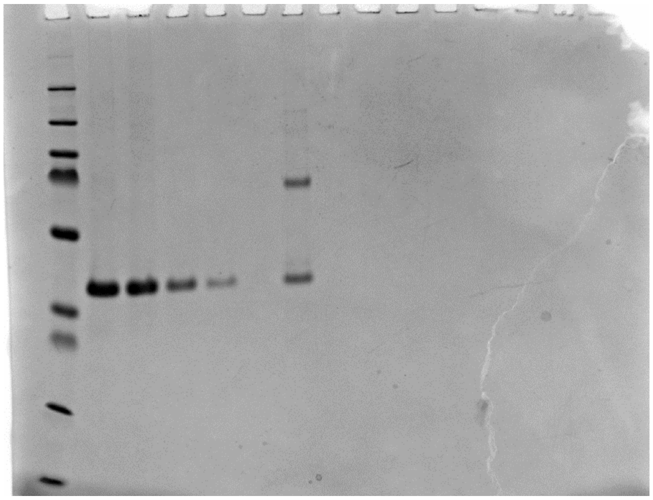

Supplementary Figure 3. Unprocessed SDS-PAGE gel images. Cropped versions appear in (a) Figure 2a and (b) Supplementary Figure 1.

Supplementary Notes

Supplementary Note 1. SpyCatcher-mi3 Sequence

MKMEELFKKHKIVAVLRANSVEEAKKKALAVFLGGVHLIEITFTVPDADTVIKELSFLKEMGAIIGAGTVTSVEQA
RKAVESGAEFIVSPHLDEEISQFAKEKGVFYMPGVMTPTELVKAMKLGHTILKLFPGEVVGPQFVKAMKGPFPN
VKFVPTGGVNLDNVCEWFKAGVLAVGVGSALVKGTPVEVAEKAKAFVEKIRGCTE

Supplementary Note 2. RBD Sequence

RVQPTESIVRFPNITNLCPFGEVFNATRFASVYAWNRKRISNCVADYSVLYNSASFSTFK
CYGVSPTKLNDLCFTNVYADSFVIRGDEVRQIAPGQTGKIADYNYKLPDDFTGCVIAWNSNNLDSK
VGGNYNYLYRLFRKSNLKPFERDISTEIYQAGSTPCNGVEGFNCYFPLQSYGFQPTNGVGYQPYRVVVLSFEL
LHAPATVCGPKKSTNLVKNKCVNF
